# Machine Learning-Based Prediction of Pulmonary Function Test Outcomes Using Oscillometry in Patients with COPD

**DOI:** 10.3390/s26206629

**Published:** 2026-10-20

**Authors:** Seyedfakhreddin Nabavi, Lennart K. A. Lundblad, Ronald J. Dandurand, Bryan A. Ross

**Affiliations:** 1 Respiratory Epidemiology and Clinical Research Unit, Centre for Outcomes Research and Evaluation, Research Institute of the McGill University Health Centre, Montreal, QC H4A 3J1, Canada; seyed.nabavi@rimuhc.ca; 2 Meakins-Christie Laboratories, Research Institute of the McGill University Health Centre, Montreal, QC H4A 3J1, Canadaronald.dandurand@mcgill.ca (R.J.D.); 3 THORASYS Thoracic Medical Systems Inc., Montreal, QC H2V 4L5, Canada; 4 Division of Respirology, Department of Medicine, McGill University, Montreal, QC H4A 3J1, Canada; 5 Montreal Chest Institute, McGill University Health Centre, Montreal, QC H4A 3J1, Canada

**Keywords:** oscillometry, lung function test, COPD, respiratory system, machine learning

## Abstract

Oscillometry has been recognized as a non-invasive method for assessing respiratory conditions and is increasingly being implemented in routine clinical practice. Currently, a full pulmonary function test (*PFT*), which requires extensive infrastructure and trained personnel, remains the standard for comprehensive lung function assessment. Furthermore, while a full *PFT* can be performed by most patients with mild chronic obstructive pulmonary disease (COPD), this can be exceedingly difficult and cumbersome to complete in patients with more advanced disease. The present study sought to investigate the relationship between lung function parameters obtained by oscillometry, a non-invasive technique requiring only quiet breathing, with the parameters obtained from the more technically demanding components of a standard *PFT,* plethysmography and diffusing capacity, in patients with mild and very severe COPD. Through Spearman analyses, the strongest associations were observed between resistance at 5 Hz (*R*_5_) and the difference in resistance between 5 Hz and 19 Hz (*R*_5_–*R*_19_), by oscillometry, with residual volume (*RV*) to total lung capacity (*TLC*) ratio *(RV/TLC*—gas trapping) and diffusing capacity of the lung for carbon monoxide (*DLco*—diffusing capacity), by *PFT* (0.69 and −0.74, respectively). To extend the clinical applicability of oscillometry, three machine learning models suitable for small datasets, Extreme Gradient Boosting (*XGBoost*), Random Forest (*RF*), and Support Vector Regression (*SVR*), were implemented to predict these key *PFT* outcomes from oscillometry metrics and baseline demographic data alone. *XGBoost* achieved the best performance, with mean absolute errors of 9.7 for *RV/TLC* and 8.1 for *DLco*. In contrast, none of the models could accurately predict *TLC*. Feature importance analysis revealed that age enhanced *RV/TLC* prediction, while *DLco* prediction was consistently influenced by the addition of body mass index (BMI). In conclusion, oscillometry has the potential to serve as complementary to *PFT* for assessing otherwise tedious, resource-intensive, and often unattainable information on gas trapping and gas exchange, especially for older or frail adults with advanced disease, while still offering high accuracy.

## 1. Introduction

Chronic obstructive pulmonary disease (COPD) is a very common progressive respiratory condition characterized by persistent and largely irreversible airflow limitation [1] and includes chronic bronchitis and emphysema. Based on modeling of risk-factor trends, the global prevalence of COPD is projected to reach around 600 million cases by 2050, marking a 23% increase from 2020, with the largest relative growth expected among women (47% vs. 9% in men) and in low- and middle-income countries [2]. The Global Initiative for Chronic Obstructive Lung Disease (GOLD), the leading non-profit international COPD organization established by the World Health Organization, categorizes COPD into four grades of airflow obstruction severity, ranging from mild (‘GOLD 1’) to very severe (‘GOLD 4’) [3]. Very severe COPD is associated with the worst clinical outcomes, with a significantly higher risk of death and frequent hospitalizations [4]. Conversely, the capacity to improve large-scale screening for mild COPD amongst individuals at risk remains a persisting challenge in the effective early diagnosis and prompt management of COPD.

The pulmonary function test (*PFT*) is a comprehensive diagnostic evaluation of the respiratory system that represents the traditional clinical standard for characterizing lung function. A full *PFT* consists of spirometry, body plethysmography, and diffusing capacity, with each of these techniques yielding unique information regarding airflow obstruction, hyperinflation, gas trapping, and gas transfer. Guidelines for performing and interpreting these tests have been established by both the European Respiratory Society (ERS) and the American Thoracic Society (ATS) [5]. In a series of forced maximal respiratory maneuvers, breath-hold maneuvers, and logistical maneuvers (transferring the patient between different apparatus), spirometry is performed first, followed by body plethysmography, and then diffusing capacity. In the interpretation of *PFT* results, the obtained values are compared with normal or predicted ranges, which are derived from large population studies of healthy individuals. These predicted values are adjusted for multiple factors including age, height, and sex [5]. Currently, spirometry, the first component of a full *PFT*, remains recommended by the latest GOLD international report for the initial diagnosis of COPD [3].

During the second component of a full *PFT*, the body plethysmography measurement, the patient is seated inside an airtight box, inhales and exhales to a particular volume through a breathing tube, and then a shutter drops across this tube. The patient then makes respiratory efforts against the closed shutter [6]. This measurement is based on Boyle’s law, which states that at a constant temperature, the volume of a given mass of gas varies inversely with pressure. Hence, lung volumes are computed by measuring the changes in pressure within the box. The body plethysmography measurement, which remains a highly challenging maneuver for patients with chronic diseases, older and/or frail patients, those with mobility issues and/or on supplemental oxygen, and in those with advanced disease, provides information about lung volumes and capacities, such as the amount of air remaining in the lungs after maximal expiration (residual volume, *RV*), and the total volume of air in the lungs after maximal inspiration (total lung capacity, *TLC*) [5]. Beyond hyperinflation (as indicated by an elevated *TLC*), these measured parameters also provide important information in identifying air trapping, determined by an increased *RV/TLC* [5]. These results can be used to inform clinical management and, if possible from a technical perspective pending the patient’s condition (i.e., capacity to complete sequential tests given the repositioning and repeated forced maneuvers necessary), can be tracked over time [7].

The third and final component of a full *PFT* is the diffusing capacity of a carbon monoxide (*DLco*) test [8], also known as the transfer factor, which provides information regarding gas exchange. This complex test (both technically and physiologically) reflects various components of lung function, including alveolar-capillary membrane diffusivity, pulmonary vascular volume, and the distribution of ventilation and perfusion [9]. Therefore, beyond the strict presence or absence of emphysema per se, ventilation-perfusion heterogeneity [10], lung hyperinflation and gas trapping, and small airways disease [11,12,13] can also affect the clinical measurement of *DLco*. *DLco* is measured using a technical single-breath inspiratory/breath-hold maneuver, where a mixture of 10% helium and 0.3% carbon monoxide is rapidly inhaled, held for 10 s, and then exhaled while the remaining carbon monoxide is measured. By comparing the fractions of inspired and expired carbon monoxide, *DLco* can be calculated. Beyond serving as a marker of disease severity, emphysema burden, and even the presence of comorbid pulmonary vascular disease, *DLco* has also been reported as a prognostic marker for exacerbation risk and mortality in patients with COPD [14].

Despite their diagnostic role and clinical relevance, body plethysmography and *DLco* are resource-intensive, requiring specialized equipment that is usually only available at larger centres, in addition to highly trained personnel and, above all, the full cooperation of patients in performing several difficult and uncomfortable breathing maneuvers. These factors unfortunately limit their accessibility, particularly in community settings and in resource-constrained environments, as well as their practicality in large proportions of the adult COPD patient population who are most likely to require the most intensive chronic disease follow-up and management.

Oscillometry has emerged as an alternative, simple and portable technique [15]. It is a non-invasive technique for evaluating the mechanical properties of the respiratory system by measuring the impedance of airflow during quiet tidal breathing. In this method, low-amplitude oscillations are applied to the airway opening (i.e., at the mouth) and then transmitted throughout the central and peripheral respiratory system, allowing the characterization of resistance and reactance, a composite metric of elastance and inertance. Oscillometry is a system identification method, where a controlled perturbation is applied to the system under investigation. The consequences of the perturbation are measured, and a mathematical model is used to link perturbation to consequence [16]. Depending on the stimulating signal used, oscillometry can assess respiratory mechanics across a spectrum of frequencies (spectral oscillometry) or within a single breath at a single frequency (within-breath or intra-breath oscillometry) [17,18]. Since no forced maneuvers are required, nor breath-hold maneuvers, nor challenging transfers (e.g., into equipment such as airtight plethysmography booths), oscillometry requires minimal technical effort and is easy to obtain at the clinical point of care. Oscillometry is therefore a very appealing solution for infants, older, frail, non-mobile patients, and those with more advanced disease, especially considering lung function testing is typically performed serially over time to monitor for disease stability and progression. The increasing clinical popularity of oscillometry is evidenced by a recently updated ERS guideline on the technical standards of oscillometry for clinical use [19]. These favourable properties of oscillometry, and the reliability and validity of its results even without a trained technician present [20], have led to several potential applications above and beyond what is possible from bulky, expensive, and non-portable *PFT* apparatus, including daily home lung function monitoring and early exacerbation detection in patients with COPD [20]. We recently demonstrated that a variant of the interrupter resistance technique, the relaxed expiratory occlusion monitor (*REOM*), a lightweight portable handheld device, could estimate airway resistance with excellent concordance and agreement with that obtained at two critical frequencies by oscillometry in patients with COPD [21]. The versatility of the oscillometry technique, its potential for widespread clinical use, and the ease of testing have cumulatively led to serious reconsideration of traditional cumbersome approaches to lung function testing and chronic disease monitoring.

The utility of oscillometry for assessing disease severity, small-airway dysfunction, exacerbation risk, and bronchodilator response in COPD populations has been established [22,23,24]. The correlation between parameters measured by oscillometry and those obtained from spirometry has been extensively studied and reported [25,26]. However, the competence of oscillometry to determine the presence of gas trapping, hyperinflation, and diffusing capacity, which have only been conventionally possible by the completion of body plethysmography and diffusing capacity, remains unclear. While there is a clear rationale that oscillometry, through the measurement of respiratory system resistance and reactance during tidal breathing, would be plausibly related to plethysmography [27], associations reported in the literature between oscillometry and several contributors to *DLco*, including ventilation-perfusion heterogeneity, small airways disease, and gas trapping [11,13,28], lend support that oscillometry may additionally plausibly have the capacity to indirectly predict this third component of a complete *PFT*.

Given this important and clinically relevant gap in knowledge, the present study sought to investigate the relationship between key parameters obtained during quiet tidal breathing using oscillometry and conventional parameters obtained from a full *PFT* in patients with COPD. Furthermore, to maximize the frontline clinical applicability of oscillometry, multiple machine learning (ML) models were implemented using basic patient demographic information alone to enhance prediction accuracy of oscillometry parameters for plethysmography and diffusing capacity measurements, with the overarching aim of determining the applicability of oscillometry as an easy and rapidly obtainable alternative, and/or complement, to the conventional full *PFT*.

## 2. Materials and Methods

### 2.1. Study Design and Eligibility Criteria

The present study used the dataset from a single-site, cross-sectional observational study conducted at the McGill University Health Centre (MUHC) [21]. Consecutive patients presenting to the subspecialized COPD clinical program at the Montreal Chest Institute (MCI) of the MUHC were screened for eligibility. The study received ethical approval from the MUHC Research Ethics Board (REB study number: 2023–9001) as well as Health Canada approval (ITA: 355823) and was registered on ClinicalTrials.gov (NCT05913323). All participants provided both written and verbal informed consent prior to enrollment.

Eligibility criteria included male or female patients aged ≥40 years, current or former smokers with a ≥10 pack-year smoking history, and a confirmed diagnosis of either mild (GOLD 1) or very severe (GOLD 4) COPD based on spirometry. Patients with comorbid asthma, on continuous home oxygen therapy, a recent acute exacerbation of COPD (within 4 weeks of testing), chronic or suppurative respiratory infections, or any absolute contraindications to performing a standard *PFT* were excluded.

### 2.2. Data Collection

All testing was conducted at the Centre for Innovative Medicine (CIM) of the MUHC during a single in-person visit, and thus for each participant, all *PFT* and oscillometry tests were performed on the same day. Since standard lung function testing (including full *PFT*) cannot precede oscillometry, oscillometry was performed first, followed by the full *PFT* [19].

Following recruitment, baseline demographic data were collected, including age, sex, body mass index (BMI), total smoking history quantified in pack-years, and disease severity classified as GOLD 1 or 4 based on spirometry performed at that visit.

Participants performed an oscillometry test (Tremoflo C-100 device, Thorasys Thoracic Medical Systems Inc., Montreal, QC, Canada). Measurements between frequencies of 5 Hz and 37 Hz were performed for the following parameters: resistance and reactance at 5 Hz (*R*_5_ and *X*_5_), resistance and reactance at 10 Hz (*R*_10_ and *X*_10_), resistance at 19 Hz (*R*_19_), and area under the reactance curve (*Ax*). For each participant, 3 technically acceptable oscillometry measurements were obtained in accordance with ERS technical standards to ensure a coefficient of variation (CV) of ≤10% for resistance at the lowest frequency, *R*_5_ [19].

As the final step of the study visit, each participant completed a full *PFT*, which included pre- and post-bronchodilator spirometry (to confirm fixed airflow obstruction and proper GOLD classification), body plethysmography, and diffusing capacity testing using standard equipment (BodyBox Plethysmography, Medisoft, MGC Diagnostics Corporation, Dinant, Belgium), in accordance with ERS/ATS recommendations and criteria [5].

### 2.3. Statistical Analysis

Demographic information and lung function results were presented either as means with standard deviation (SD) or as medians with interquartile range (IQR) as appropriate based on data distribution. Normality was assessed using the Shapiro–Wilk test. Following this, Spearman correlation coefficients were used to assess the relationship between each oscillometry parameter and key measurements obtained from body plethysmography and diffusing capacity testing. Statistical significance was set at *p* < 0.05. All analyses were performed using MATLAB (Natick, MA, USA) version 9.13.0 (R2022b).

### 2.4. Machine Learning Models

Three machine learning models suitable for small datasets [29,30,31], Extreme Gradient Boosting (*XGBoost*), Random Forest (*RF*), and Support Vector Regression (*SVR*), were utilized to assess the prediction of body plethysmography and diffusing capacity *PFT* parameters using oscillometry-recorded metrics with basic baseline patient characteristics. Categorical variables (e.g., sex) were one-hot encoded, and all features were standardized prior to model training [32].

Given the small sample size, and to maximize the use of the available data, model performance was evaluated using leave-one-out cross-validation (LOOCV), in which each patient served once as the held-out test case. Hyperparameters were selected via grid search using LOOCV with Mean Absolute Error (MAE) as the optimization metric. Model performance was reported using several conventional comparative metrics, namely MAE, Root Mean Squared Error (RMSE), and the Coefficient of Determination (R^2^) score. Feature importance analysis was additionally conducted using SHapley Additive exPlanations (SHAP) [33] to identify the most influential predictors contributing to each model’s performance.

## 3. Results

**Figure 1:**
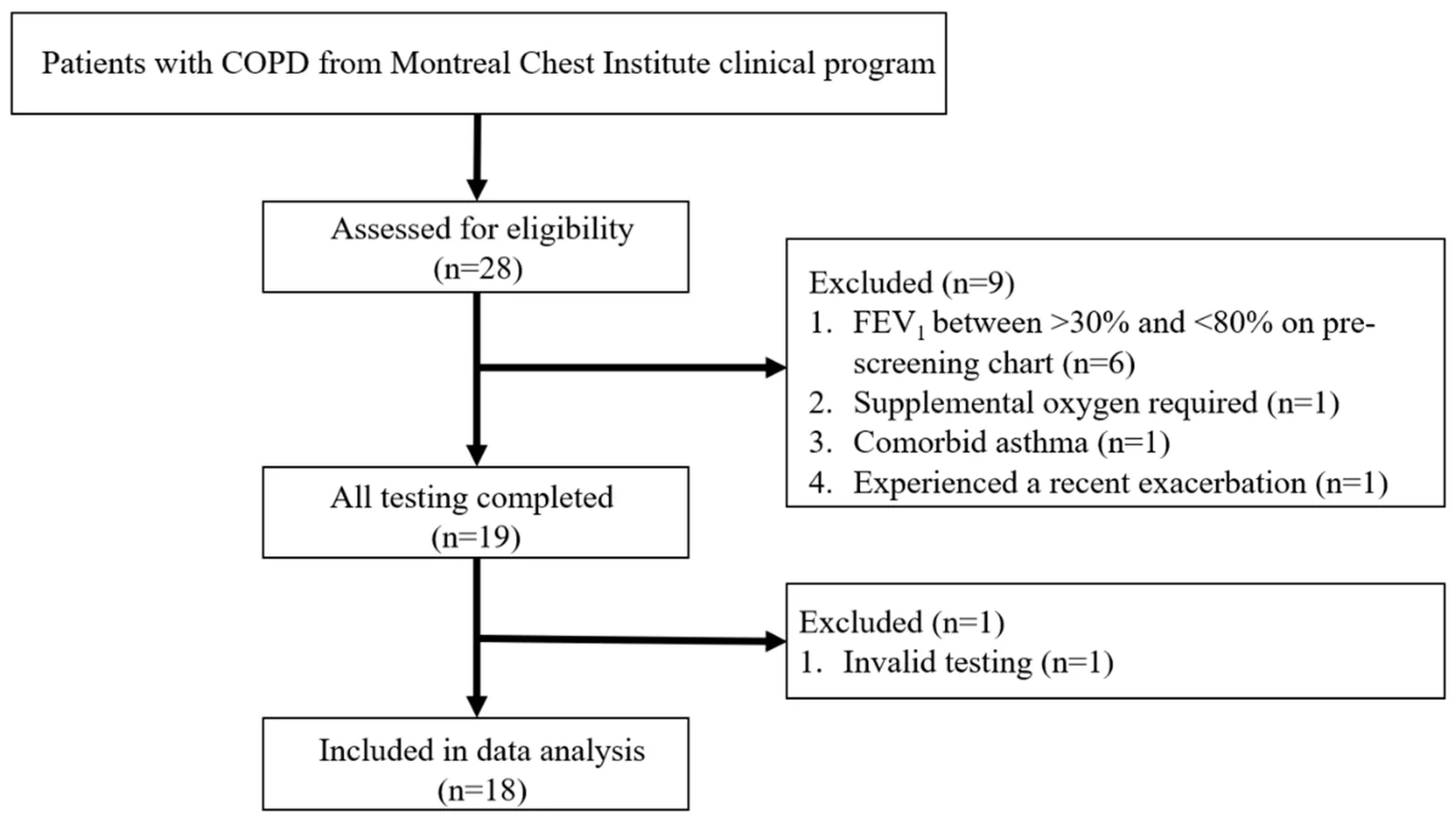
Flow diagram of study participants. COPD, chronic obstructive pulmonary disease; FEV_1_, forced expiratory volume in the first second.

The flow diagram of study participants is presented in Figure 1. Of the 19 participants recruited and tested, 18 were included in the final analysis. Baseline demographic characteristics for all participants and by group are presented in Table 1. Nine participants had GOLD 1 (mild) COPD, and 9 had GOLD 4 (very severe) COPD. Participants in the GOLD 1 COPD group were older and had a considerably higher BMI than those in the GOLD 4 COPD group, with (by definition) considerably different inter-group airflow obstruction severity (mean *FEV*_1_ exceeding 90% of predicted, compared with mean *FEV*_1_ of approximately 24% of predicted, respectively). The groups were similar in terms of sex distribution and reported comparable cumulative cigarette smoking histories.

**Figure 2:**
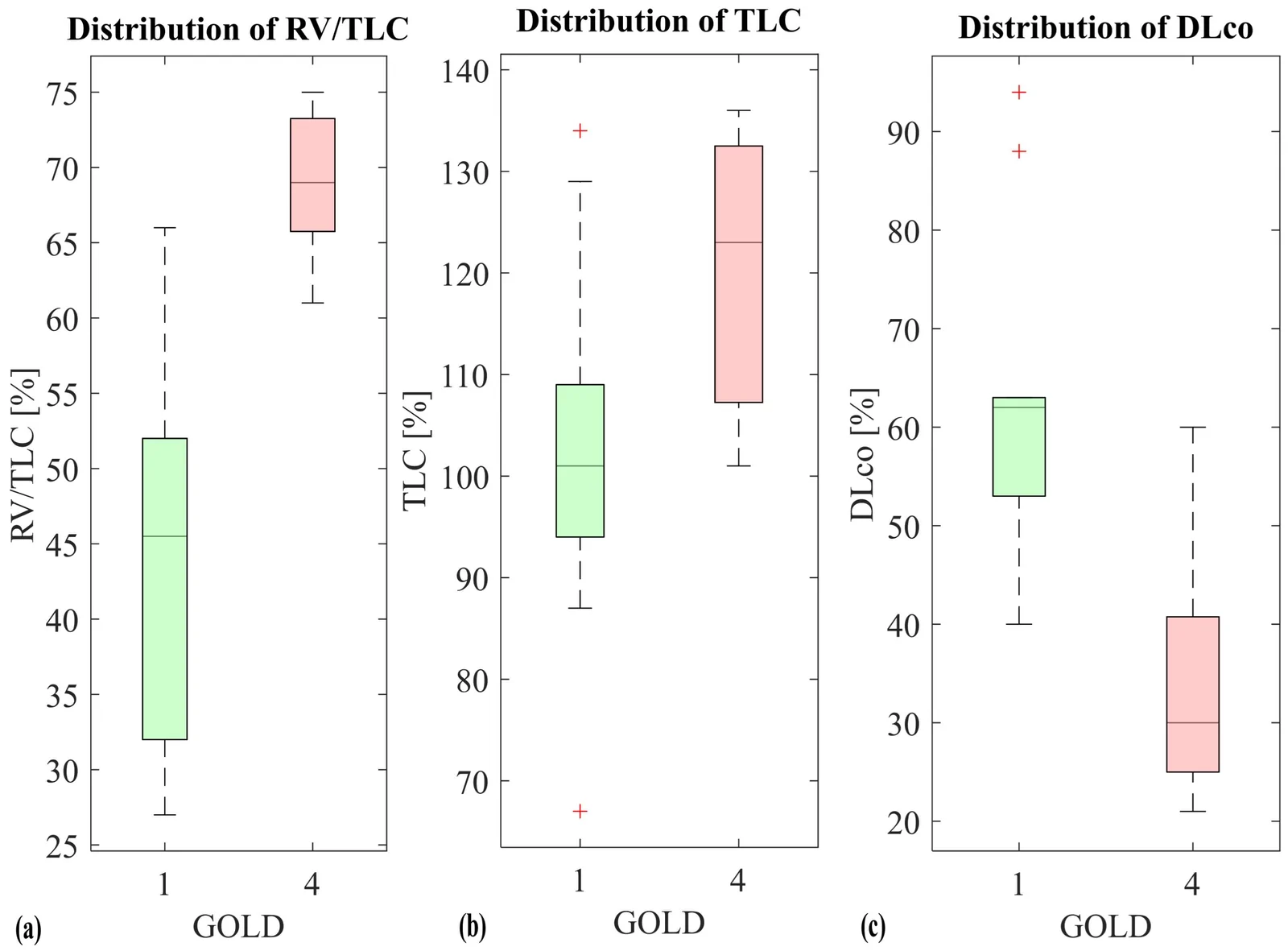
Pulmonary function testing (PFT) measurements: (**a**) Ratio of residual volume to total lung capacity (RV/TLC), (**b**) total lung capacity (TLC), and (**c**) diffusion capacity for carbon monoxide (DLco). The horizontal line within each box represents the median, and the lower and upper edges of the box represent the 25th and 75th percentiles (interquartile range, IQR). Whiskers extend to the most extreme data points within 1.5 × IQR from the box edges. Red crosses (+) indicate outliers, defined as values beyond 1.5 × IQR.

Body plethysmography and diffusing capacity measurements from *PFT*—*RV/TLC*, *TLC*, and *DLco*—are presented by subgroup in Figure 2. Gas trapping (*RV/TLC*) and hyperinflation (*TLC*) are more pronounced in the GOLD 4 COPD group. As previously described [34], gas trapping is more discordant between groups than hyperinflation. *DLco* was very low in the GOLD 4 COPD group, with a median value of only 28% of predicted.

**Figure 3:**
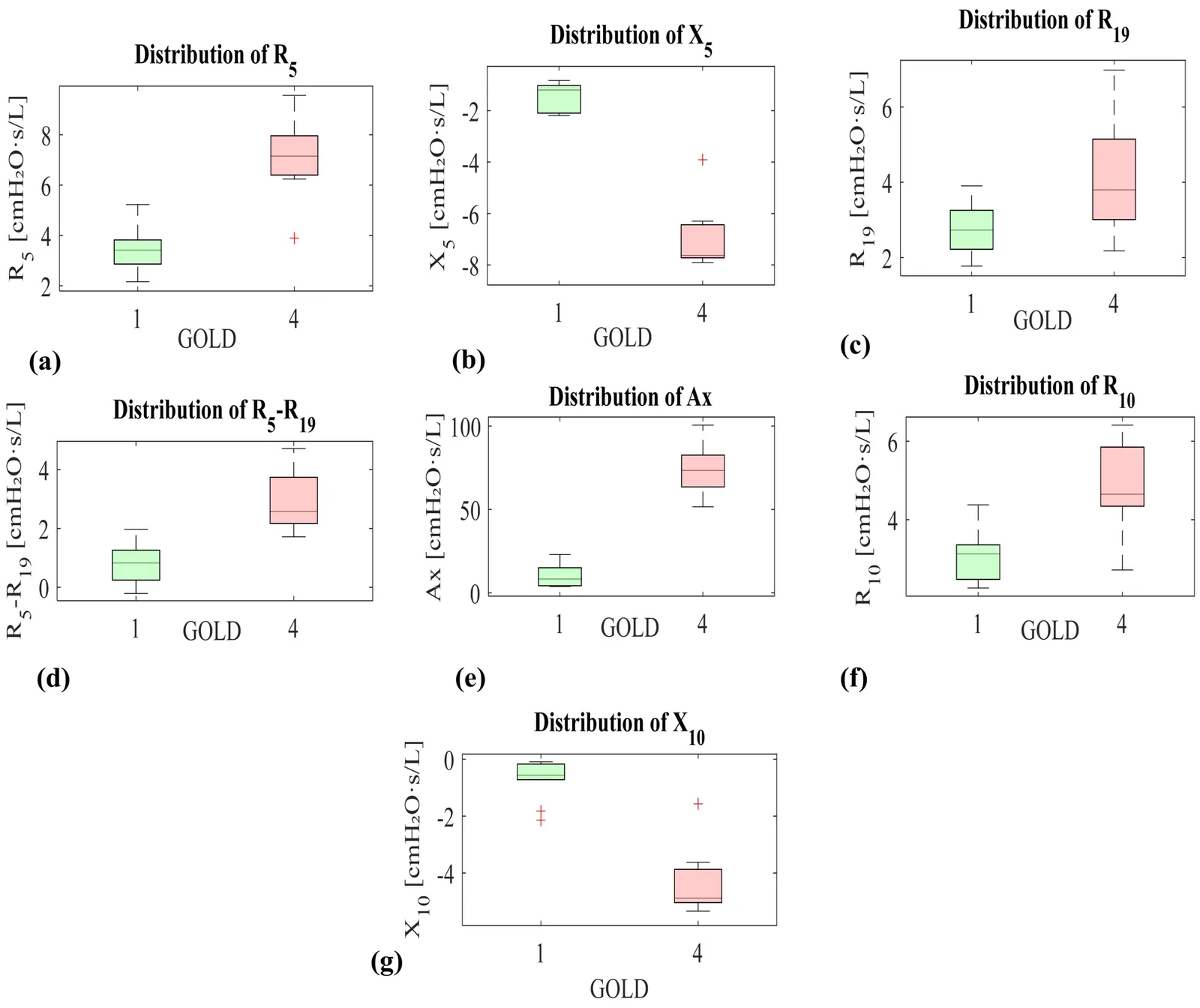
Distribution of oscillometry parameters between GOLD 1 and GOLD 4: (**a**) Resistance at 5 Hz (R_5_), (**b**) reactance at 5 Hz (X_5_), (**c**) resistance at 19 Hz (R_19_), (**d**) difference in resistance between 5 Hz and 19 Hz (R_5_–R_19_), (**e**) area under the reactance curve (Ax), (**f**) resistance at 10 Hz (R_10_), and (**g**) reactance at 10 Hz (X_10_). The horizontal line within each box represents the median, and the lower and upper edges of the box represent the 25th and 75th percentiles (interquartile range, IQR). Whiskers extend to the most extreme data points within 1.5 × IQR from the box edges. Red crosses (+) indicate outliers, defined as values beyond 1.5 × IQR.

Each measured oscillometry parameter is presented, by subgroup, in Figure 3. When compared to individuals with GOLD 1 COPD, in individuals with GOLD 4 COPD, all resistance parameters are higher (more severe), all reactance parameters are more negative (more severe), and the ‘area’-based parameter (*Ax*) is larger in amplitude (more severe) when compared to individuals with GOLD 1 COPD.

**Figure 4:**
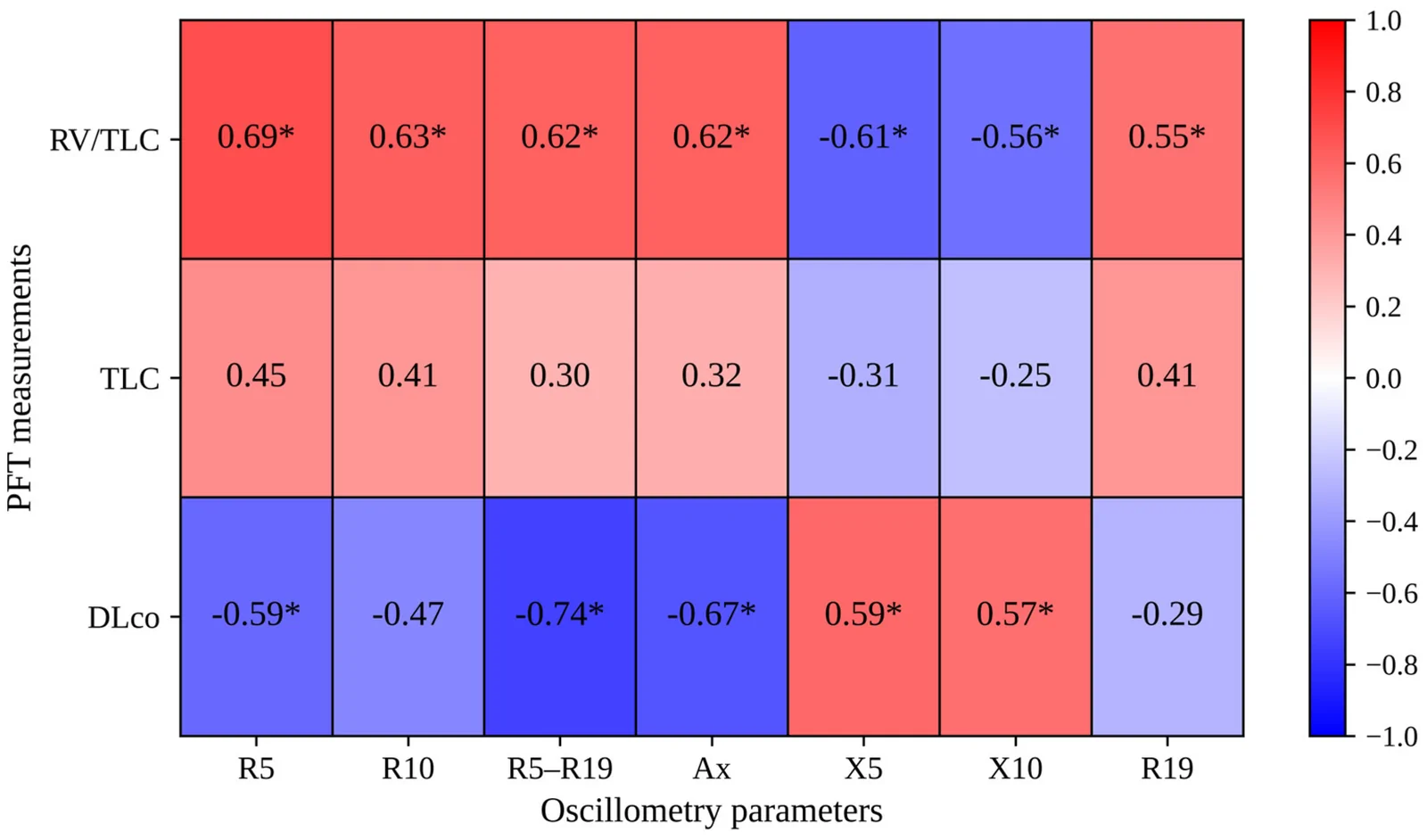
Computed Spearman correlations between PFT measurements and oscillometry parameters (* indicates *p* < 0.05).

The computed Spearman correlation coefficients across oscillometry parameters and *PFT* (body plethysmography and diffusing capacity) outcomes are summarized as a heatmap in Figure 4. For both *RV/TLC* and *TLC* (for which *higher* values indicate *more severe* gas trapping and hyperinflation), positive correlations were observed with resistance, and negative correlations were observed with reactance parameters. In contrast, for *DLco* (for which *lower* values indicate *more severe* gas transfer abnormalities), similar general relationships of opposite magnitude were observed. The strongest associations were observed between *R*_5_ and *RV/TLC* (ρ = 0.69) and between *R*_5_*–R*_19_ and *DLco* (ρ = −0.74). Conversely, oscillometry parameters exhibited only modest associations with *TLC*, with the highest correlation coefficient of 0.45 observed with *R*_5_.

**Figure 5:**
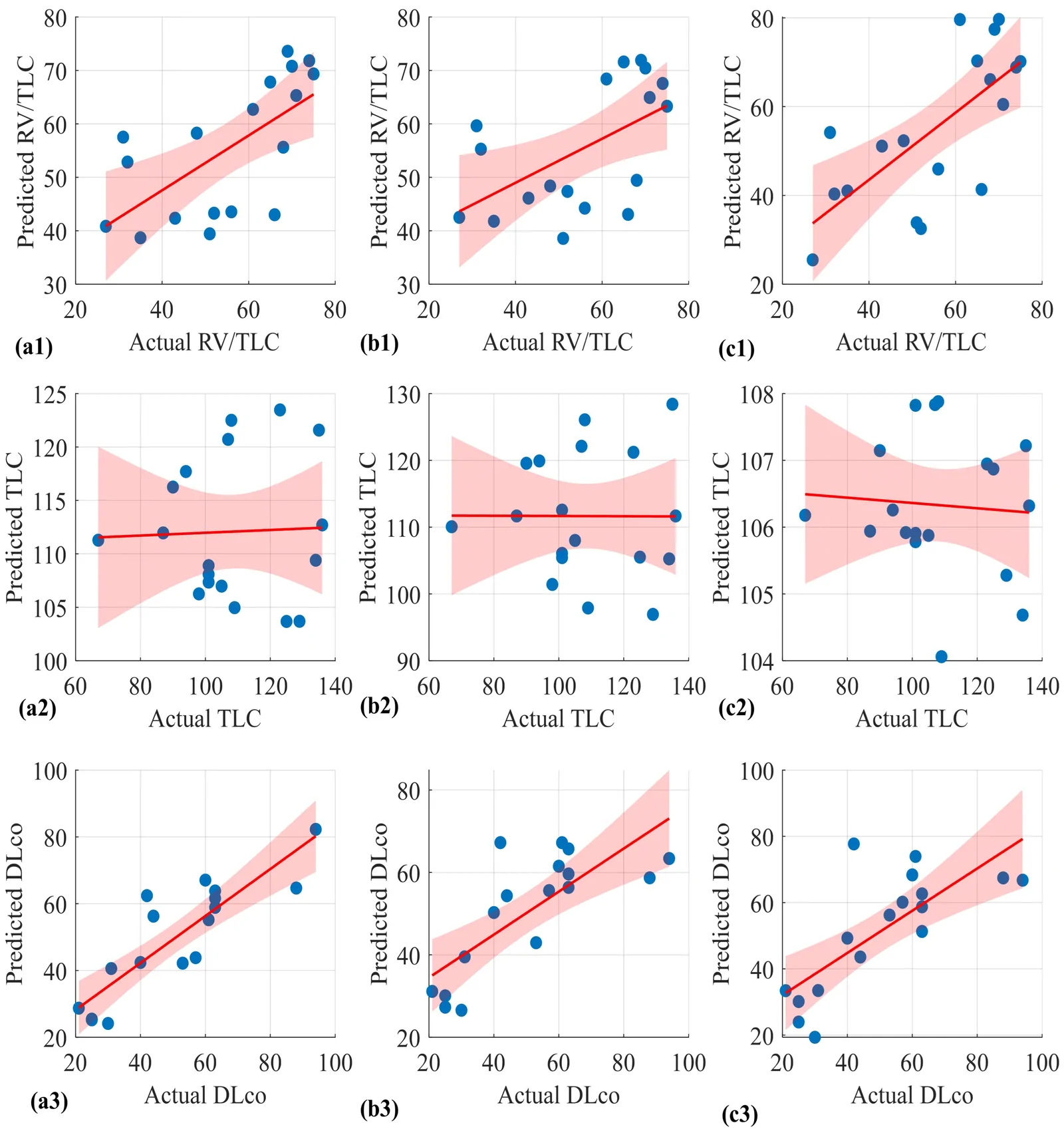
Predicted versus actual PFT measurements using (**a**) XGBoost, (**b**) random forest, and (**c**) SVR. Indices **1**, **2**, and **3** correspond to RV/TLC, TLC, and DLco, respectively. The shaded regions represent the 95% confidence intervals.

The *XGBoost*, *RF*, and *SVR* models were applied to predict each of the *PFT* measurements from oscillometry parameters with the addition of simple and easily obtained demographic variables. The predicted versus actual values for the body plethysmography and diffusing capacity *PFT* measurements, estimated by each standalone model, are presented in Figure 5. The majority of model predictions appear to fall within the 95% confidence intervals for *RV/TLC*, as well as (in a model-dependent manner) for *DLco*. None of the implemented ML models for *TLC* appear to demonstrate a clear or consistent correlation between predicted and actual values.

The predictive performance of *XGBoost*, *RF*, and *SVR* models across *RV/TLC*, *TLC*, and *DLco* is summarized in Table 2. Amongst the models, *XGBoost* demonstrated the highest accuracy for *RV/TLC* (R^2^ = 0.44, Spearman ρ = 0.72, *p* = 0.001) and *DLco* (R^2^ = 0.76, Spearman ρ = 0.85, *p* = 0.001). In contrast, predictions of *TLC* showed weak performance across all models, reflected by negative or near-zero R^2^ values and non-significant rank associations.

**Figure 6:**
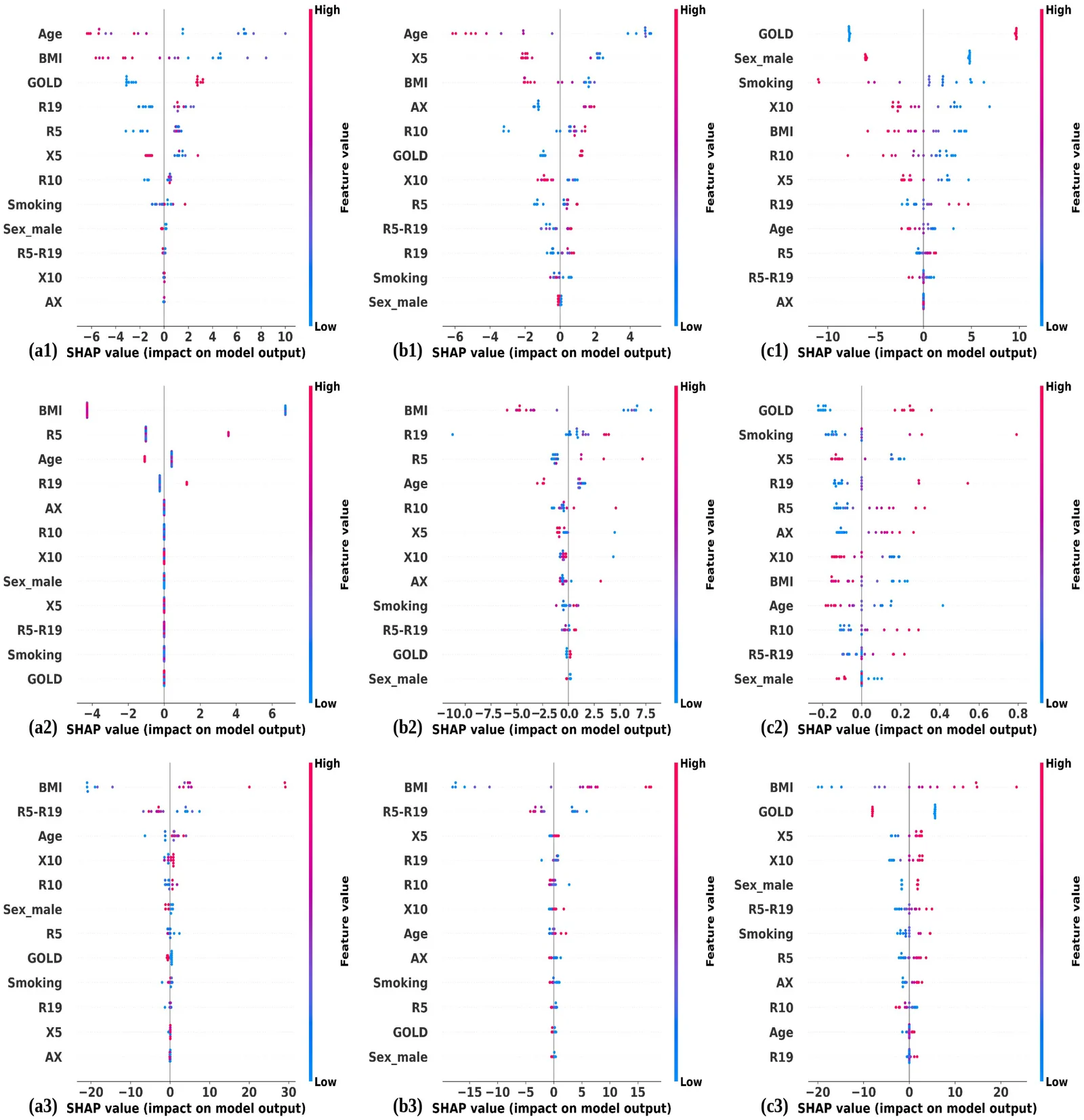
Relative impact of input parameters on the performance of models. SHAP value plots for (**a**) XGBoost, (**b**) random forest, and (**c**) SVR. Indices **1**, **2**, and **3** correspond to RV/TLC, TLC, and DLco, respectively.

The relative contribution of each input parameter to the predictions of the developed ML models, as determined using SHAP, is illustrated in Figure 6. Notably, demographic variables contributed substantially to model predictions alongside oscillometry parameters. In the *XGBoost* model predicting *RV/TLC*, age and BMI were ranked as the first and second most influential features, respectively, followed by GOLD severity and oscillometry parameters such as *R*_19_ and *R*_5_. In all three models predicting *DLco*, BMI was consistently the most influential feature, while *R*_5_*–R*_19_ ranked second in both the *XGBoost* and *RF* models, consistent with its strong correlation with *DLco* (Figure 4). Conversely, *TLC* predictions were influenced by fewer and less consistent features across models, in line with the weaker predictive performance observed for *TLC* (Table 2). It is important to note that, given the small sample size, the ranking of the most influential predictors may be unstable and should be interpreted only as exploratory.

Since GOLD severity may carry substantial information about *PFT* outcomes, particularly given the inclusion of two extreme disease groups, an ablation analysis excluding this variable was performed. This resulted in only a marginal increase in MAE (from 9.70 to 9.85 for *RV/TLC* and from 8.10 to 8.20 for *DLco*), indicating that its contribution to model performance was minimal. Moreover, since spirometry is routinely performed as the first step in diagnosing COPD, GOLD severity is readily available for patients with COPD; hence, including it as an input feature does not add complexity to the proposed approach or limit its clinical applicability.

## 4. Discussion

The present study evaluated the relationship between each oscillometry parameter and body plethysmography and diffusing capacity, two cumbersome and resource-intensive components of a full *PFT*, with the aim of determining whether an easily obtainable and comfortable point-of-care test could serve as a complementary or alternative method for these widely deployed and inflexible tests. While only moderate associations were observed between oscillometry (namely, *R*_5_) and *TLC*, the prevailing marker of hyperinflation, strong associations were observed between *R*_5_, *X*_5_, *R*_10_, *R*_5_*–R*_19_, and *Ax* with *RV/TLC,* the prevailing marker of gas trapping. All oscillometry parameters except for reactance parameters were inversely correlated with *DLco*, among which *R*_5_*–R*_19_ and *Ax* exhibited the strongest Spearman correlation coefficients (ρ = −0.74 and −0.67, respectively). While oscillometry parameters may be directly associated with plethysmography outcomes, their relationship with *DLco* is likely to be more indirect, reflecting the broader effects of disease severity and small airway dysfunction [13]. Following the testing of three distinct ML models (i.e., *XGBoost*, *RF*, *SVR*) for prediction of plethysmography and diffusing capacity, *RV/TLC* was effectively predicted, with a minimum MAE of 9.7, by leveraging the *XGBoost* model. This model also exhibited high predictive performance with statistically significant results for *DLco* (MAE of 8.1). Consistent with the statistical analyses, none of the implemented models were able to predict *TLC* with high accuracy. This mainly reflects a fundamental limitation of oscillometry in assessing hyperinflation, rather than failure of the developed models. Finally, when the relative impact of input features was analyzed, the best-performing models for predicting *RV/TLC* were *XGBoost* and *RF*, in which age emerged as the most influential feature, while all models consistently identified BMI as a primary input feature for *DLco* prediction.

While the relatively low association between *TLC* and all oscillometry parameters and predictive models in patients with COPD was unexpected from the standpoint of the use of *TLC* in conventional clinical practice, a similarly weak association between *TLC* and clinically important COPD outcomes, in comparison with *RV/TLC,* has been extensively studied and reported previously in the literature. When the mechanistic association between computed tomography-obtained diaphragm dome height (a marker of diaphragm flattening in COPD and itself closely associated with lung function, exercise performance, disease burden and future exacerbation risk) and conventional *PFT* markers of hyperinflation and gas trapping was investigated in 304 participants with mild and moderate COPD, significant associations were observed only with *RV/TLC* and not with *TLC* [35]. Furthermore, when full *PFT* data was analyzed across 7157 visits in 1988 patients with mild to very severe COPD, the closest relationships with markers of disease severity on spirometry were observed with *RV/TLC* and not with *TLC* [36]. Indeed, in the present study, and in previous work [34], gas trapping (*RV/TLC*) was more discordant between disease severity groups than was hyperinflation (*TLC*) and, as such, *RV/TLC* appears to be better suited as a disease severity discriminatory test than *TLC*. By extension, the observed capacity of oscillometry to accurately predict *RV/TLC* in the present study is a particular and relevant strength.

Gas exchange occurs at the level of the alveolar-capillary interface. In patients with COPD, prolonged inhaled noxious exposures can lead to dysfunction and destruction of large amounts of alveolar-capillary units even in mild disease [10]. Parenchymal destruction also occurs by this same mechanism, which affects the capacity of the smaller airways, that do not possess cartilaginous support, to remain patent. These two phenomena (alveolar-capillary destruction and parenchymal destruction) affect ventilation-perfusion matching, can be seen on imaging or pathologically from lung tissue biopsy or post-mortem [37], and in essence collectively represent ‘emphysema’ in the COPD patient population. Interestingly, small airway collapse and destruction can even precede the appearance of emphysema on imaging [38]. In the present study, a close association between *DLco*, a marker of gas transfer, and *R*_5_*–R*_19_, a marker of the resistance of the small peripheral airways, was observed and is likely explained by the common mechanism reflecting the burden of emphysema, which leads to concomitant alveolar-capillary destruction (affecting gas exchange) as well as a reduced tethering effect on the smaller airways, which do not otherwise have cartilaginous support to remain open (affecting small airway resistance) [39].

While the present study is one of the first of its kind to explore not only associations but also predictions of *PFT* parameters using oscillometry, these findings are consistent with other studies that focused on COPD and non-COPD chronic respiratory disease populations. For instance, Wu et al. [40] reported significant associations between *Ax*, *X*_5_, and *R*_5_*–R*_19_ and *RV/TLC* in patients with interstitial lung disease and hypersensitivity pneumonitis, concluding in that study that oscillometry may help distinguish between different types of respiratory diseases. Similarly, Savushkina et al. [41] demonstrated that increases in *Ax* and *R*_5_*–R*_19_ were associated with reduced *DLco* following COVID-19. ML approaches leveraging oscillometry parameters have been increasingly applied to strengthen the clinical utility of oscillometry in respiratory disease diagnosis [42,43]. In a recent study, Nemeth et al. [44] demonstrated the effectiveness of oscillometry integrated with ML models for classifying respiratory categories among COPD, interstitial lung disease (ILD), obesity hypoventilation syndrome (OHS), and healthy controls. The proposed framework achieved a test accuracy of 98.5% in distinguishing tidal expiratory flow limitation between healthy individuals and patients with COPD. Despite advances in integrating oscillometry with ML, prior efforts have primarily emphasized disease classification. Hence, the present study is novel in that it applies ML models to directly predict body plethysmography and diffusing capacity *PFT* measurements from oscillometry parameters, in a manner that is further enhanced by inputting basic patient demographic information.

Age and BMI were particularly relevant in improving the accuracy of ML model prediction. The contributions of age to improving *RV/TLC* prediction likely reflect the fact that advancing age promotes gas trapping and elevates *RV/TLC* [45]. In contrast, the effect of BMI in contributing to *DLco* prediction performance, and the effect more broadly in the COPD patient population, is more complicated and nuanced. While obesity is prevalent in industrialized nations around the world [46] and is associated with poorer health outcomes, in COPD there can be a unique ‘bimodal’ distribution of BMI. A lower, rather than a higher, BMI is associated with the emphysema ‘phenotype’ [47], and by extension, a low BMI has consistently been demonstrated to be prognostic of mortality in COPD [48]. Therefore, the relationship between BMI and *DLco* observed in the present study is likely a reflection of their shared consequence of emphysema [49].

Finally, regarding the respective predictive capacity of the individual ML models tested, *XGBoost* achieved the highest predictive performance. This is likely due to its inherent gradient boosting framework, which sequentially corrects prediction errors and effectively captures complex nonlinear relationships among oscillometry as well as demographic information. Furthermore, the inherent regularization of *XGBoost* may have contributed to its superior performance compared to *RF* and *SVR* [50].

The scientific and clinical relevance of this study is noteworthy. In the setting of increasingly limited healthcare resources and the minimum necessary infrastructure required to perform a full *PFT*, in comparison to the growing global prevalence and burden of COPD, these results support the applicability of oscillometry as a non-invasive measurement technique that can be readily applied as a diagnostic tool without the need for substantial fixed laboratory equipment or specialized personnel, while also providing complementary physiological and clinically relevant insights on gas trapping and gas exchange that are typically only accessible through a full *PFT*. The integration of oscillometry with an ML-based model can enable clinicians to obtain actionable respiratory insights directly at the point of care, without requiring patients to undergo a conventional *PFT*. Accordingly, such an approach has the potential to streamline clinical-decision making, reduce patient participant burden, and facilitate more frequent and accessible respiratory assessments in routine clinical practice.

Notably, inter-device variability in oscillometry measurements can be considerable. For instance, Dandurand et al. tested six oscillometry devices using mechanical test loads that simulate the respiratory system [51]. They observed that measurement differences between devices increased with higher impedance values. Hence, to minimize such bias when different oscillometry data are used to train ML models, a harmonization approach, such as including the device indicator as an input feature to the models, should be considered.

Despite its many strengths, the present study also has important limitations. It focused on patients with mild and very severe COPD to intentionally capture the extremes of disease, while those with moderate and severe disease were not included. As the relationship between *PFT* and oscillometry parameters may be non-linear across GOLD grades, the present models may not be applicable to patients with moderate (GOLD 2) or severe (GOLD 3) COPD. Furthermore, despite efforts to maintain a balanced distribution of baseline demographic features between participant groups, the limited dataset may have restricted the full capacity of the developed ML models.

## 5. Conclusions and Future Directions

This study investigated the associations between oscillometry parameters and standard body plethysmography and diffusing capacity *PFT* measurements in patients with COPD. Oscillometry parameters demonstrated stronger associations with gas trapping and diffusing capacity. Leveraging these physiological relationships, we implemented multiple ML models incorporating oscillometry parameters together with baseline demographic information. This simple and clinically applicable approach predicted body plethysmography and diffusing capacity measurements. Notably, none of the models could predict *TLC*, which likely reflects a fundamental limitation of oscillometry in assessing hyperinflation rather than a failure of the models. These preliminary findings highlight the potential of oscillometry as a practical, accessible complement to conventional *PFT* that can be readily performed at the point of care, facilitate clinical decision-making, and reduce patient and health system burden.

Future work should focus on implementing the proposed technique in larger datasets and more diverse populations, including patients across all GOLD grades. As oscillometry outputs vary between devices, external validation through independent, multi-centre cohorts using different oscillometry devices is required before this approach can be considered for routine clinical use. Such heterogeneous datasets would enable the application of more advanced modeling approaches, including deep learning, with the potential to further improve prediction accuracy for full *PFT* outcomes. Ultimately, this method may support point-of-care assessment and impact frontline clinical care.

## Figures and Tables

**Table 1:** Participant baseline demographic information.

	All Participants	GOLD 1	GOLD 4
**Participants, No.**	18	9	9
**Age, years**	66.3 ± 8.1	71.8 ± 5.8	60.8 ± 6.1
**BMI, Kg/m^2^**	25.1 ± 6.8	26.6 ± 2.6	23.5 ± 9.3
**Smoking History, pack-years**	34.8 ± 24.2	29.4 (18.8)	40.2 ± 28.7
**FEV_1_, Liters**	1.5 ± 0.9	2.4 ± 0.5	0.7 ± 0.2
**FEV_1_, % predicted**	58.0 ± 35.4	91.4 ± 11.4	24.6 ± 4.8
**FVC, Liters**	3.1 ± 1.2	3.91 ± 0.9	2.3 ± 0.7
**FVC, % predicted**	89.7 ± 29.0	114.6 ± 15.4	64.8 ± 12.7
**Female Sex, No. (%)**	10 (55.6)	5 (55.6)	5 (55.6)
**Male Sex, No. (%)**	8 (44.4)	4 (44.4)	4 (44.4)

Spirometry values are post-bronchodilator.

**Table 2:** Predictive performance of machine learning models for pulmonary function outcomes.

Model	Output	RMSE (95% CI)	MAE (95% CI)	R^2^ Score (95% CI)	Spearman ρ (95% CI)	*p*-Value
XGBoost	RV/TLC	12.1 [8.2, 15.6]	9.7 [6.4, 13.1]	0.44 [0.18, 0.74]	0.72 [0.37, 0.90]	0.001 *
	TLC	22.7 [14.5, 30.2]	17.8 [11.9, 24.5]	0.01 [−0.52, 0.13]	0.11 [−0.38, 0.52]	0.651
	DLco	10.4 [6.6, 13.6]	8.1 [5.1, 11.5]	0.76 [0.39, 0.89]	0.85 [0.56, 0.96]	0.001 *
RF	RV/TLC	13.4 [9.2, 17.1]	10.9 [7.5, 14.6]	0.31 [0.11, 0.65]	0.60 [0.21, 0.79]	0.001 *
	TLC	23.1 [15.6, 29.5]	18.4 [12.3, 24.9]	−0.03 [−0.75, 0.18]	0.01 [−0.41, 0.52]	0.723
	DLco	13.5 [7.2, 18.5]	9.8 [5.9, 14.5]	0.57 [0.16, 0.79]	0.75 [0.30, 0.94]	0.001 *
SVR	RV/TLC	12.3 [8.1, 15.6]	9.8 [6.4, 13.4]	0.42 [0.12, 0.78]	0.71 [0.37, 0.88]	0.001 *
	TLC	23.7 [14.4, 32.1]	17.3 [10.8, 25.1]	−0.01 [−0.65, 0.02]	0.13 [−0.34, 0.52]	0.615
	DLco	13.8 [7.8, 19.2]	9.9 [5.9, 14.9]	0.55 [0.30, 0.79]	0.71 [0.24, 0.92]	0.008 *

* Indicates statistical significance.

## Data Availability

The data supporting the findings of this study are available from the corresponding author upon reasonable request, subject to approval by the Research Ethics Board and compliance with university policies.

## References

[1] Pauwels R.A., Rabe K.F. (2004). Burden and Clinical Features of Chronic Obstructive Pulmonary Disease (COPD). Lancet.

[2] Boers E., Barrett M., Su J.G., Benjafield A.V., Sinha S., Kaye L., Zar H.J., Vuong V., Tellez D., Gondalia R. (2023). Global Burden of Chronic Obstructive Pulmonary Disease through 2050. JAMA Netw. Open..

[3] GOLD Report. Global Strategy for Prevention, Diagnosis and Management of COPD. https://goldcopd.org/2026-gold-report-and-pocket-guide/.

[4] Brat K., Svoboda M., Zatloukal J., Plutinsky M., Volakova E., Popelkova P., Novotna B., Dvorak T., Koblizek V. (2021). The Relation between Clinical Phenotypes, GOLD Groups/Stages and Mortality in COPD Patients—A Prospective Multicenter Study. Int. J. Chron. Obstruct. Pulmon. Dis..

[5] Stanojevic S., Kaminsky D.A., Miller M.R., Thompson B., Aliverti A., Barjaktarevic I., Cooper B.G., Culver B., Derom E., Hall G.L. (2022). ERS/ATS Technical Standard on Interpretive Strategies for Routine Lung Function Tests. Eur. Respir. J..

[6] Criée C.P., Sorichter S., Smith H.J., Kardos P., Merget R., Heise D., Berdel D., Köhler D., Magnussen H., Marek W. (2011). Body Plethysmography--Its Principles and Clinical Use. Respir. Med..

[7] Haynes J.M., Kaminsky D.A., Ruppel G.L. (2023). The Role of Pulmonary Function Testing in the Diagnosis and Management of COPD. Respir. Care.

[8] Graham B.L., Brusasco V., Burgos F., Cooper B.G., Jensen R., Kendrick A., MacIntyre N.R., Thompson B.R., Wanger J. (2018). 2017 ERS/ATS standards for single-breath carbon monoxide uptake in the lung. Eur. Respir. J..

[9] Wagner P.D., Dantzker D.R., Dueck R., Clausen J.L., West J.B. (1977). Ventilation-Perfusion Inequality in Chronic Obstructive Pulmonary Disease. J. Clin. Investig..

[10] Ross B.A., Brotto A.R., Fuhr D.P., Phillips D.B., van Diepen S., Bryan T.L., Stickland M.K. (2020). The Supine Position Improves but Does Not Normalize the Blunted Pulmonary Capillary Blood Volume Response to Exercise in Mild COPD. J. Appl. Physiol..

[11] Zhou K., Wu F., Lu L., Tang G., Deng Z., Dai C., Zhao N., Wan Q., Peng J., Wu X. (2025). Association between Impaired Diffusion Capacity and Small Airway Dysfunction: A Cross-Sectional Study. ERJ Open Res..

[12] Bhatt S.P., Criner G.J., Han M.K., Bon J., Sciurba F.C. (2026). Prevalence of the Phenotype of Lung Hyperinflation and Minimal Emphysema in COPD. Am. J. Respir. Crit. Care Med..

[13] Yao S., Huang L., Chen B., Ji L., Zheng J., Gao Y. (2026). Impulse Oscillometry and Traditional Pulmonary Function Testing: Correlation, Advances and Clinical Implications. BMJ Open Respir. Res..

[14] Ni Y., Yu Y., Dai R., Shi G. (2021). Diffusing Capacity in Chronic Obstructive Pulmonary Disease Assessment: A Meta-Analysis. Chron. Respir. Dis..

[15] DuBois A.B., Brody A.W., Lewis D.H., Burgess B.F. (1956). Oscillation Mechanics of Lungs and Chest in Man. J. Appl. Physiol..

[16] Bates J.H.T., Irvin C.G., Farré R., Hantos Z. (2011). Oscillation Mechanics of the Respiratory System. Compr. Physiol..

[17] Hantos Z. (2021). Intra-Breath Oscillometry for Assessing Respiratory Outcomes. Curr. Opin. Physiol..

[18] Dellacà R.L., Santus P., Aliverti A., Stevenson N., Centanni S., Macklem P.T., Pedotti A., Calverley P.M.A. (2004). Detection of Expiratory Flow Limitation in COPD Using the Forced Oscillation Technique. Eur. Respir. J..

[19] King G.G., Bates J., Berger K.I., Calverley P., de Melo P.L., Dellacà R.L., Farré R., Hall G.L., Ioan I., Irvin C.G. (2020). Technical Standards for Respiratory Oscillometry. Eur. Respir. J..

[20] Walker P.P., Pompilio P.P., Zanaboni P., Bergmo T.S., Prikk K., Malinovschi A., Montserrat J.M., Middlemass J., Šonc S., Munaro G. (2018). Telemonitoring in Chronic Obstructive Pulmonary Disease (CHROMED). A Randomized Clinical Trial. Am. J. Respir. Crit. Care Med..

[21] Coutu F.-A., Malaeb D., Iorio O.C., Nabavi S., McFarland T., Lundblad L.K.A., Dandurand R.J., Gottfried S.B., Ross B.A. (2025). Measuring Lung Mechanics in Patients with COPD Using the Handheld Portable Rapid Expiratory Occlusion Monitor (REOM): A Cross-Sectional Study. Physiol. Rep..

[22] Park H., Lee H.J., Lee H.W., Park T.Y., Heo E.Y., Kim D.K., Lee J.-K. (2024). Diagnosis and Evaluation of Small Airway Disease and COPD Using Impulse Oscillometry. Sci. Rep..

[23] Lu L., Wu F., Tang G., Wan Q., Deng Z., Peng J., Dai C., Zhou K., Wu X., Yu S. (2025). Associations of Small Airway Dysfunction Assessed by Impulse Oscillometry with Lung Function Decline and Exacerbations in Participants with Chronic Obstructive Pulmonary Disease: A Prospective Cohort Study in China. Respir. Med..

[24] Saadeh C., Saadeh C., Cross B., Gaylor M., Griffith M. (2015). Advantage of Impulse Oscillometry over Spirometry to Diagnose Chronic Obstructive Pulmonary Disease and Monitor Pulmonary Responses to Bronchodilators: An Observational Study. SAGE Open Med..

[25] Piorunek T., Kostrzewska M., Cofta S., Batura-Gabryel H., Andrzejczak P., Bogdański P., Wysocka E., Pokorski M. (2014). Impulse Oscillometry in the Diagnosis of Airway Resistance in Chronic Obstructive Pulmonary Disease. Allergens and Airway Hyperreactivity.

[26] Lu L., Wan Q., Tang G., Wu F., Deng Z., Peng J., Dai C., Zhou K., Wu X., Yu S. (2025). New Classifications of COPD Severity Based on Impulse Oscillometry: A SAIO Grade Base on ECOPD Cohort in China. Arch. Bronconeumol..

[27] Budin E.C., Vladimir I., Râjnoveanu R.M., Huțanu D., Vultur M.A., Muarginean C., Muntean S.T., Ianoși E.S., Jimborean G., Cotoi O.S. (2025). Correlation Between Body Plethysmography and Impulse Oscillometry Across Obstructive and Restrictive Lung Diseases: Evidence from an Adult Pilot Study Cohort. Diagnostics.

[28] Eddy R.L., Westcott A., Maksym G.N., Parraga G., Dandurand R.J. (2019). Oscillometry and Pulmonary Magnetic Resonance Imaging in Asthma and COPD. Physiol. Rep..

[29] Zou M., Jiang W.-G., Qin Q.-H., Liu Y.-C., Li M.-L. (2022). Optimized XGBoost Model with Small Dataset for Predicting Relative Density of Ti-6Al-4V Parts Manufactured by Selective Laser Melting. Materials.

[30] Basak D., Pal S., Patranabis D.C. (2007). Support Vector Regression. Neural Inf. Process.-Lett. Rev..

[31] Han S., Williamson B.D., Fong Y. (2021). Improving Random Forest Predictions in Small Datasets from Two-Phase Sampling Designs. BMC Med. Inform. Decis. Mak..

[32] Shanker M., Hu M.Y., Hung M.S. (1996). Effect of Data Standardization on Neural Network Training. Omega.

[33] Nohara Y., Matsumoto K., Soejima H., Nakashima N. (2022). Explanation of Machine Learning Models Using Shapley Additive Explanation and Application for Real Data in Hospital. Comput. Methods Programs Biomed..

[34] Smith B.M., Hoffman E.A., Basner R.C., Kawut S.M., Kalhan R., Barr R.G. (2014). Not All Measures of Hyperinflation Are Created Equal: Lung Structure and Clinical Correlates of Gas Trapping and Hyperexpansion in COPD: The Multi-Ethnic Study of Atherosclerosis (MESA) COPD Study. Chest.

[35] Beydoun S.A., Iorio O.C., Genkin D., Li P.Z., Doiron D., Maltais F., Bourbeau J., Tan W., Sin D.D., Aaron S.D. (2025). Utility of Diaphragm Dome Height as a Marker of Operational Lung Volume Changes, Disease Burden and Exacerbations in Patients with Mild-to-Moderate COPD: An Observational Study within the CanCOLD Cohort. ERJ Open Res..

[36] Alter P., Orszag J., Kellerer C., Kahnert K., Speicher T., Watz H., Bals R., Welte T., Vogelmeier C.F., Jörres R.A. (2020). Prediction of Air Trapping or Pulmonary Hyperinflation by Forced Spirometry in COPD Patients: Results from COSYCONET. ERJ Open Res..

[37] Ley-Zaporozhan J., Ley S., Eberhardt R., Weinheimer O., Fink C., Puderbach M., Eichinger M., Herth F., Kauczor H.-U. (2007). Assessment of the Relationship between Lung Parenchymal Destruction and Impaired Pulmonary Perfusion on a Lobar Level in Patients with Emphysema. Eur. J. Radiol..

[38] McDonough J.E., Yuan R., Suzuki M., Seyednejad N., Elliott W.M., Sanchez P.G., Wright A.C., Gefter W.B., Litzky L., Coxson H.O. (2011). Small-Airway Obstruction and Emphysema in Chronic Obstructive Pulmonary Disease. N. Engl. J. Med..

[39] O’Donnell D.E., Laveneziana P., Webb K., Neder J.A. (2014). Chronic Obstructive Pulmonary Disease: Clinical Integrative Physiology. Clin. Chest Med..

[40] Wu J.K.Y., Xu J.J.-N., Numakura T., Ryan C.M., McInnis M.C., Binnie M., Shapera S., Fisher J.H., Hantos Z., Chow C.-W. (2025). Standard Pulmonary Function Tests and Respiratory Oscillometry Patterns in Hypersensitivity Pneumonitis and Idiopathic Pulmonary Fibrosis. BMJ Open Respir. Res..

[41] Savushkina O.I., Astanin P.A., Nekludova G.V., Avdeev S.N., Zaytsev A.A. (2023). The Possibilities of Impulse Oscillometry in the Diagnosis of the Lung Function Disorders after COVID-19. Ter. Arkhiv.

[42] Xu W.-J., Shang W.-Y., Feng J.-M., Song X.-Y., Li L.-Y., Xie X.-P., Wang Y.-M., Liang B.-M. (2024). Machine Learning for Accurate Detection of Small Airway Dysfunction-Related Respiratory Changes: An Observational Study. Respir. Res..

[43] do Amaral J.L., de Sá Sousa C., de Oliveira Ribeiro C., de Sá P., Lopes A.J., de Melo P. (2025). A Diagnostic Support System Based on Interpretable Machine Learning and Oscillometry for Accurate Diagnosis of Respiratory Dysfunction in Silicosis. bioRxiv.

[44] Németh Á.S., Makan G., Baglyas S., Lorx A., Chow C.-W., Wu J.K.Y., Dandurand R.J., Hantos Z., Kalmár G. (2025). Pattern Recognition in Intra-Breath Oscillometry Measurements. IEEE Access.

[45] Shin T.R., Oh Y.-M., Park J.H., Lee K.S., Oh S., Kang D.R., Sheen S., Seo J.B., Yoo K.H., Lee J.-H. (2015). The Prognostic Value of Residual Volume/Total Lung Capacity in Patients with Chronic Obstructive Pulmonary Disease. J. Korean Med. Sci..

[46] Goodarzynejad H., Meaney C., Brauer P., Greiver M., Moineddin R., Monavvari A.A. (2022). Recent Trends in Adult Body Mass Index and Prevalence of Excess Weight: Data from the Canadian Primary Care Sentinel Surveillance Network. Can. Fam. Physician..

[47] Kim Y.W., Lee C.-H., Hwang H.-G., Kim Y.-I., Kim D.K., Oh Y.-M., Lee S.H., Kim K.U., Lee S.-D. (2019). Resting Hyperinflation and Emphysema on the Clinical Course of COPD. Sci. Rep..

[48] Celli B.R., Cote C.G., Marin J.M., Casanova C., de Oca M., Mendez R.A., Pinto Plata V., Cabral H.J. (2004). The Body-Mass Index, Airflow Obstruction, Dyspnea, and Exercise Capacity Index in Chronic Obstructive Pulmonary Disease. N. Engl. J. Med..

[49] Guerra S., Sherrill D.L., Bobadilla A., Martinez F.D., Barbee R.A. (2002). The Relation of Body Mass Index to Asthma, Chronic Bronchitis, and Emphysema. Chest.

[50] Santoro D., Ciano T., Ferrara M. (2024). A Comparison between Machine and Deep Learning Models on High Stationarity Data. Sci. Rep..

[51] Dandurand R.J., Lavoie J.-P., Lands L.C., Hantos Z. (2019). Comparison of Oscillometry Devices Using Active Mechanical Test Loads. ERJ Open Res..

